# Reduced Expression of Membrane Complement Regulatory Protein CD59 on Leukocytes following Lung Transplantation

**DOI:** 10.3389/fimmu.2017.02008

**Published:** 2018-01-22

**Authors:** Laura A. Michielsen, Kevin Budding, Daniël Drop, Ed A. van de Graaf, Tineke Kardol-Hoefnagel, Marianne C. Verhaar, Arjan D. van Zuilen, Henny G. Otten

**Affiliations:** ^1^Department of Nephrology and Hypertension, University Medical Center Utrecht, Utrecht University, Utrecht, Netherlands; ^2^Laboratory of Translational Immunology, University Medical Center Utrecht, Utrecht University, Utrecht, Netherlands; ^3^Department of Respiratory Medicine, University Medical Center Utrecht, Utrecht University, Utrecht, Netherlands

**Keywords:** complement, CD59, lung transplantation, lymphocyte biology, accommodation

## Abstract

Cellular protection against undesired effects of complement activation is provided by expression of membrane-bound complement regulatory proteins including CD59. This protein prevents membrane attack complex formation and is considered to be involved in graft accommodation. Also, CD59 downregulates CD4+ and CD8+ T-cell activation and proliferation. It is unknown whether CD59 expression is affected by transplantation. The aim of this study was to evaluate the quantitative CD59 antigen expression on distinct leukocyte subsets following lung transplantation (*n* = 26) and to investigate whether this differs from pretransplantation (*n* = 9). The results show that CD59 expression on leukocytes is significantly lower posttransplantation compared with healthy controls (*p* = 0.002) and pretransplantation (*p* < 0.0001). Moreover, the CD59 expression diminishes posttransplantation on all distinct lymphocyte subsets (*p* < 0.02). This effect appeared to be specific for CD59 since the expression of other surface markers remained stable or inclined following transplantation. The highest antigen expression posttransplantation was observed on CD4+ T cells and monocytes (*p* ≤ 0.002). These findings show that CD59 expression on leukocytes diminishes posttransplantation, which could result in decreased resistance against complement and enhanced T-cell activation. If such reduction in CD59 expression also occurs on endothelial cells from the transplanted organ, this could lead to a change into a prothrombotic and proinflammatory phenotype.

## Introduction

CD59 is a glycosylphosphatidylinositol-anchored complement regulatory protein that interferes with membrane attack complex (MAC) formation by blocking the binding of C9 to C5b-C8. MAC formation may result in cell lysis, whereas sublytic levels can result in cellular activation, altered proliferation, and the release of cyto- and chemokines through interference with certain signal transduction pathways (1–3). For example, sublytic MAC levels on the endothelium can enhance alloreactive T-cell activation through upregulation of noncanonical nuclear factor-kappaB (NF-κB) resulting in a proinflammatory genetic profile (4). Furthermore, the MAC promotes a prothrombotic phenotype by inducing platelet activation and release of von Willebrand factor and procoagulent plasma membrane vesicles from endothelial cells (5–7). Apart from preventing formation of the MAC, CD59 may directly interfere with T-cell activation and proliferation upon exogenous antigen binding (8).

CD59 is widely expressed in almost all tissues and on all circulating cells, though expression levels differ greatly between cell types (9, 10). Endothelial cells exhibit much higher CD59 expression levels compared with peripheral blood mononuclear cells (PBMCs) and within PBMC subsets, including in CD4+ and CD8+ T cell subsets, differences are also observed (11–13). However, these data are based on relative differences in mean fluorescence intensity (MFI) values whereas information on absolute CD59 antigen density on these cells is lacking.

Complement regulation is considered to play an important role in maintaining long-term allograft survival by inducing resistance against antibody-mediated complement-dependent cell lysis, a process called accommodation (14–16). Our lab has identified a single-nucleotide polymorphism (SNP) in the promoter region of CD59 (rs147788946) that is associated with lower expression levels on lung donor endothelial cells and monocytes, but not on lymphocytes, and decreased resistance against complement-mediated cell lysis. The presence of this SNP configuration in lung donors was also associated with a higher incidence of bronchiolitis obliterans syndrome (BOS) and a tendency toward impaired long-term patient survival (17). These results support the hypothesis that CD59 expression levels on the donor endothelium correlate with graft survival. However, little is known on CD59 expression levels posttransplantation when patients are subjected to immunosuppressive therapy. Because posttransplant endothelial cells were not available since transbronchial biopsies are not routinely being performed, we used PBMCs as a model system. The aim of this study was to evaluate the quantitative CD59 antigen expression on distinct leukocyte subsets following lung transplantation (LTx) and to investigate whether this differs from pretransplant CD59 antigen expression.

## Patients and Methods

### Patients and Sample Collection

Twenty-nine patients who underwent LTx in the UMC Utrecht between April 2004 and June 2012 were included in this study based on sample availability. From these patients, 19 patients were exclusively included in posttransplant measurements, 3 solely in pretransplant measurements, and of 7 both pre- and posttransplant samples were included. In addition, nine healthy controls were also included in this study. Standard immunosuppressive therapy posttransplantation consisted of tacrolimus, mycophenolate mofetil (MMF), and prednisone with basiliximab induction therapy. Written informed consent was obtained from all patients and healthy controls. This study was approved by the Medical Research Ethics Committee of the UMC Utrecht (protocol METC 06-144) and performed in accordance with the Declaration of Helsinki.

Patient blood samples were routinely collected directly before transplantation and monthly during the first-year posttransplantation. For this study, we used samples that were taken between months 2 and 5 posttransplantation. Because of limited cell numbers, we used samples taken at different time points. PBMCs were isolated from heparin blood using Ficoll-Paque Plus (GE Healthcare, Little Chalfont, UK). Samples were frozen in RPMI medium (Thermo Fischer, Waltham, MA, USA) supplemented with 20% fetal bovine serum (FBS; Bodinco, Alkmaar, The Netherlands) and 10% dimethyl sulfoxide (Sigma-Aldrich, St. Louis, MO, USA) and preserved in liquid nitrogen until analysis.

### Quantification of CD59 Expression

Peripheral blood mononuclear cells were rapidly thawed in a warm water bath (37°C) and added to warm RPMI medium with 20% FBS, centrifuged and dissolved in phosphate-buffered saline (PBS). Cells were divided for anti-CD59 (BioLegend, San Diego, CA, USA) staining or isotype IgG2a (BioLegend) staining in a 2:1 ratio and incubated for 30 min at 4°C in the dark. The median number of cells stained for CD59 was 1.7 × 10^6^ (IQR 1.1 × 10^6^; 2.2 × 10^6^) and for isotype IgG2a control 8.4 × 10^5^ (IQR 6.0 × 10^5^; 1.2 × 10^6^). Following washing, cells and QIFIKIT beads (Dako, Glostrup, Denmark) were simultaneously stained with a saturating concentration of goat anti-mouse IgG FITC to determine absolute CD59 expression quantified as antibody-binding capacity (ABC). The QIFIKIT kit contains five bead populations with a distinct and known amount of monoclonal mouse antibody bound per microsphere bead. By constructing a calibration curve based on fluorescence intensity of the different populations plotted against their known antibody density, CD59 expression on the PBMCs can be interpolated based on their MFI. The specific antibody-binding capacity (SABC) is calculated by subtracting the calculated ABC for corresponding isotype controls from the anti-CD59 ABC. We also determined the estimated CD59 expression on lung donor endothelial cells obtained at time of transplantation (Supplementary Material).

### Leukocyte Subsets Staining and Complement-Mediated Cell Lysis

To study CD59 expression on different leukocyte subsets and to relate this to resistance to complement-mediated cell lysis, cells stained for CD59 or isotype control were equally divided over three different tubes designated as follows: (1) no HLA class I antibodies, no serum; (2) HLA class I antibodies + serum heat inactivated (HI), and (3) HLA class I antibodies + serum. Antibody panels to identify lymphocyte subsets were added to each tube and tubes 2 and 3 were concomitantly incubated with 3 µl of 500 µg/ml HLA class I antibody clone W6/32 (ITK Diagnostics, Uithoorn, The Netherlands) for 30 min at 4°C in the dark. This dosage of antibodies correlated with 50% cell lysis on average in previous dose-finding experiments (17). Pooled human serum of two healthy volunteers was diluted 1:5 with veronal saline buffer (Lonza, Basel, Switzerland). 25 µl PBS was added to tube 1; 25 µl serum HI 1:5 was added to tube 2, and 25 µl of serum 1:5 was added to tube 3. Following incubation at 37°C for 15 min, cells were washed with annexin-V binding buffer and incubated with annexin-V PE (BD Biosciences, Franklin Lakes, NJ, USA) and 7AAD (BD Biosciences) for 15 min. Samples were measured on a BD FACS LSR II with 10-color detection (BD Biosciences).

### Influence of Immunosuppressive Drugs and Immune Activation on CD59 Expression

To assess the effect of immunosuppressive drugs and immune activation on CD59 expression, we used whole blood and PBMCs of six healthy volunteers. Whole blood samples were spiked with: tacrolimus 10 ng/ml (Selleckchem, Houston, TX, USA), mycophenolic acid 2.5 µg/ml (Sigma-Aldrich), prednisolone 21-acetate 150 ng/ml (Santa Cruz Biotechnology, Dallas, TX, USA), a combination of these drugs or PBS as a negative control. Samples were incubated for 24 h at 37°C, 5% CO_2_. This incubation time was selected based on preliminary time course experiments, showing a maximum effect after 24 h of incubation. Following incubation, the erythrocytes were lysed with lysing solution (BD Biosciences) and stained for flow cytometry analysis. PBMCs of the same donors were cultured for 24 h at 37°C, 5% CO_2_ in the presence or absence of human T activator CD3/CD28 dynabeads (Invitrogen, Waltham, MA, USA). Subsequently, CD59 expression on T cells was measured with flow cytometry analyses.

### Antibodies

Leukocyte subsets were identified using an antibody panel containing CD45-PO (Life Technologies) for lymphocytes, CD3-AF 700 (BioLegend) for T cells, CD14-PeCy7 (BioLegend) for monocytes, CD19-BV711 (BioLegend) for B cells, and CD16/CD56-APC (eBioscience, BD Biosciences) for NK cells. T cell subsets were distinguished by CD3-AF 700 (BioLegend), CD4-BV711 (BioLegend), CD8-PeCy7 (BD), CD27-APC-eF780 (eBioscience, San Diego, CA, USA), and CD45RO-PB (BioLegend). Each sample was also stained with isotype-matched control monoclonal antibodies for spectral compensation and to correct for background fluorescence. Because of the extensive antibody panel, some antibodies were measured in the same fluorescence channel, e.g., CD4 and CD19, CD8 and CD14, and CD16 and CD56. A representative example of the gating strategy to identify the different lymphocyte subsets is provided in Figure S1 in Supplementary Material.

### Statistics

All data were analyzed with GraphPad Prism 7 (GraphPad Software Inc., San Diego, CA, USA). The Mann–Whitney test and Wilcoxon matched-pairs signed rank test, as appropriate, were used to compare groups. A *p*-value of <0.05 was considered to be statistically significant.

## Results

### Patient Demographics

Patient and transplant characteristics are summarized in Table 1. The percentage of patients developing BOS or acute rejection was slightly higher in the patients that were included in posttransplant analyses, though this was not significant. Nine healthy controls were also included in this study, the majority of these were woman (*n* = 7), and their age was comparable to the lung transplant recipients (median 48 years, IQR 44; 59). The proportion of the different leukocyte subsets did not notably differ between months 2 and 5 posttransplantation (Figure S2 in Supplementary Material).

**Table 1:** Patient and transplant characteristics.

	Pretransplantation	Posttransplantation
**Recipient**		
Total number	10	26
Gender, male	4 (40%)	11 (42%)
Median age (years)	53 (45; 59)	50 (38; 55)
Primary disease		
COPD	6 (60%)	11 (42%)
Cystic fibrosis	2 (20%)	10 (38%)
Interstitial lung disease	2 (20%)	5 (19%)
Infection		
CMV high risk	2 (20%)	5 (19%)
EBV high risk	1 (10%)	2 (8%)
Clinical complications		
BOS	3 (30%)	13 (50%)
Episode of AR	1 (10%)	7 (27%)
Patient death	4 (40%)	10 (38%)
Type of transplantation		
Unilateral	5 (50%)	6 (23%)
Bilateral	5 (50%)	20 (77%)
Graft ischemic time (min)		
Right lung	222 (178; 265)	210 (180; 247)
Left lung	338 (248; 395)	331 (278; 389)
**Donor**		
Gender, male	4 (40%)	11 (42%)
Median age (years)	49 (45; 57)	48 (39; 56)
Smoking, yes	4 (40%)	9 (35%)
Donor type		
DBD	8 (80%)	22 (85%)
DCD	2 (20%)	4 (15%)

*Overview of patient and transplant characteristics. Data are presented as number (percentages) or as median (interquartile range) as appropriate. Seven patients were included both in pre- and posttransplant analyses*.

*AR, acute rejection; BOS, bronchiolitis obliterans syndrome; CMV, cytomegalovirus; COPD, chronic obstructive pulmonary disease; DBD, donation after brain death; DCD, donation after cardiac death; EBV, Epstein–Barr virus*.

*EBV of CMV high risk: CMV/EBV negative recipient and CMV/EBV positive donor*.

### CD59 Expression on Leukocytes Is Markedly Lower in Lung Transplant Patients

**Figure 1:**
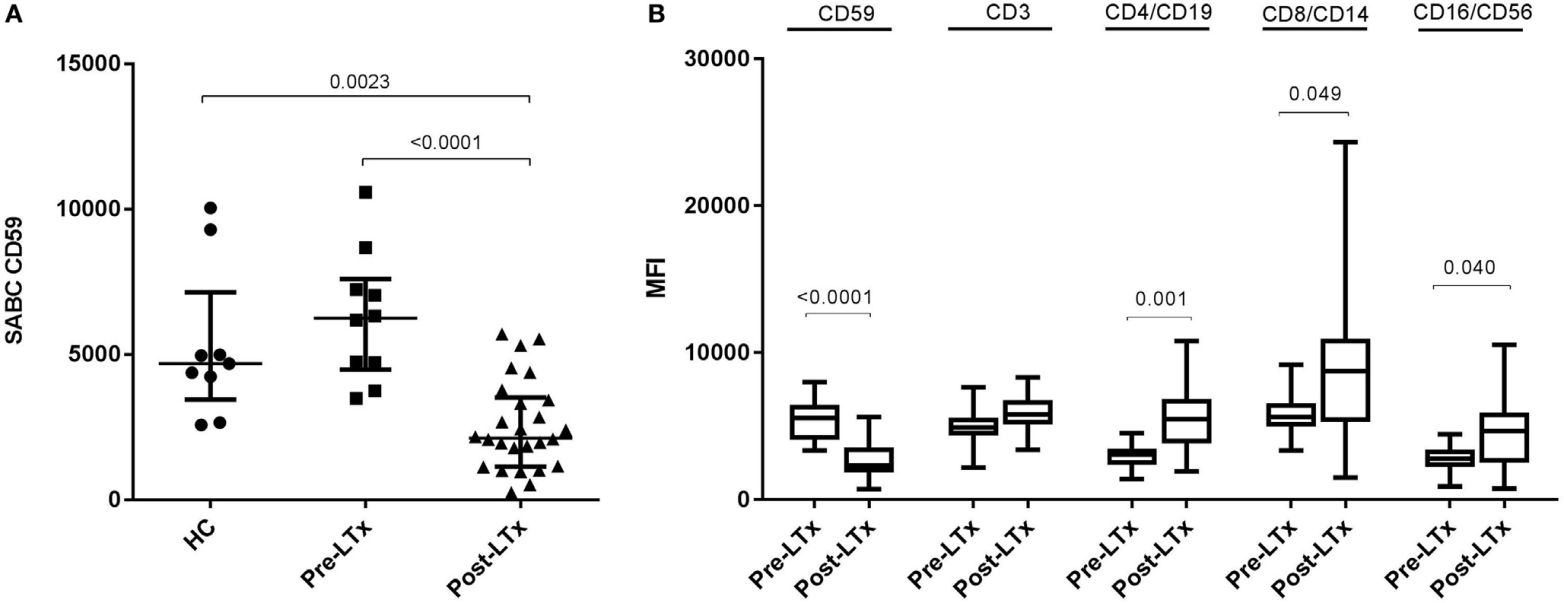
CD59 expression on leukocytes of lung transplant patients is lower compared with healthy controls (HC) and patients with end-stage lung disease. **(A)** Depiction of specific antibody-binding capacity (SABC) of CD59 on leukocytes measured by flow cytometry analysis and corrected for background fluorescence. Lung transplant patients show significant lower CD59 expression on leukocytes compared with healthy controls (*p* = 0.002) and pretransplantation (*p* < 0.0001). Data represent median and interquartile range; symbols indicate individual values. **(B)** Comparison of mean fluorescence intensity (MFI) levels of different surface expression markers on CD45+ cells shows that while CD59 expression decreases posttransplantation, other markers remain stable or tend to incline. Data are presented as box-and-whisker plots with boxes covering the interquartile range and median displayed within, and whiskers displaying minimum and maximum values. Data were analyzed with Mann–Whitney test.

**Figure 2:**
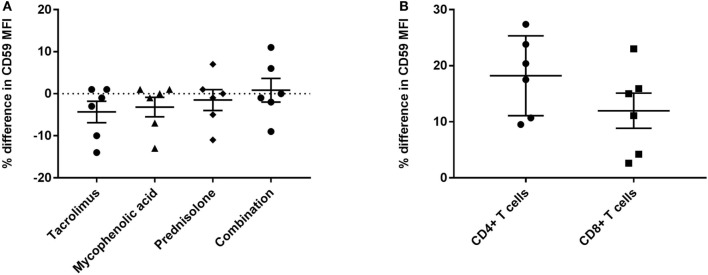
CD59 expression is not markedly affected by immunosuppressive drugs or immune activation. **(A)** Percentage of difference in CD59 mean fluorescence intensity (MFI) on CD45+ lymphocytes for whole blood samples incubated with tacrolimus 10 ng/ml, mycophenolic acid 2.5 μg/ml, prednisolone 150 ng/ml or a combination of these drugs compared with phosphate-buffered saline. **(B)** Percentage of difference in CD59 MFI on CD4+ and CD8+ T cells for lymphocytes stimulated with CD3/CD28 beads compared with unstimulated lymphocytes. Data represent mean and standard error of the mean; symbols indicate individual values.

**Figure 3:**
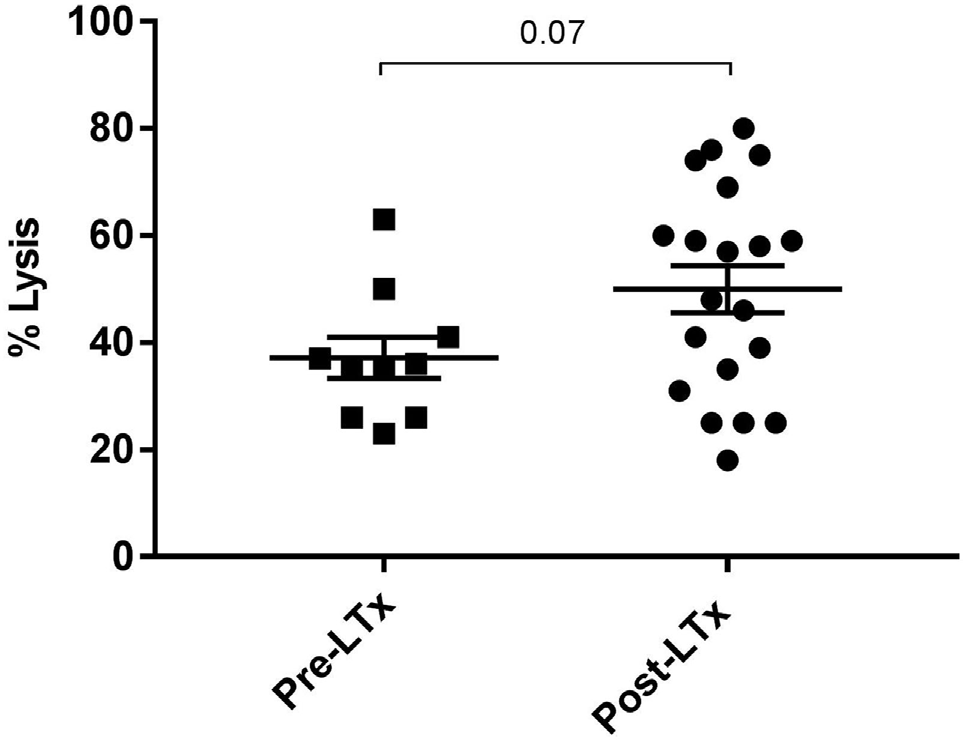
Complement-mediated cell lysis pre- and posttransplantation. The mean percentage of cell lysis in samples with anti-HLA class I antibody and serum compared with cells that were not incubated with anti-HLA class I antibody was 37% for the pretransplant samples and 50% for the posttransplant samples (*p* = 0.07). Data represent mean and standard error of the mean; symbols indicate individual values. Data were analyzed with the unpaired *t*-test.

First, we compared quantitative expression of CD59 on leukocytes of healthy controls to pre- and post LTx. The results indicate that the expression of CD59 did not differ significantly between healthy controls and pretransplant samples, while it was significantly lower posttransplantation compared with pretransplantation (*p* < 0.0001) and healthy controls (*p* = 0.002) (Figure 1A). The number of CD59 surface molecules post LTx was on average 2.9 times lower compared with pretransplantation. Next, we looked at MFI levels of different surface expression markers on leukocytes that were used to identify distinct subsets, to investigate whether this lowered expression was a general effect. Unlike for CD59 expression, CD45+ cells showed a significant increase in the combined expression of CD4/CD19, CD8/CD14, and CD16/CD56 and a tendency toward higher CD3 MFI levels following LTx (Figure 1B). In comparison, the expression of CD59 on lung donor endothelial cells obtained at time of transplantation is notably higher compared with leukocytes (Figure S3 in Supplementary Material). To investigate whether CD59 expression is affected by immunosuppressive therapy, we incubated whole blood samples with different immunosuppressive drugs. In this short-term experiment, incubation with immunosuppressive drugs did not result in a striking decrease in CD59 expression on lymphocytes compared with PBS (Figure 2A). Furthermore, activation of lymphocytes with CD3/CD28 dynabeads did only result in a modest increase in CD59 expression on T cells (Figure 2B). To test whether CD59 expression levels were related to sensitivity to complement-mediated cell lysis, PBMCs were incubated with a suboptimal dose anti-HLA class I antibody and serum as a source of complement. The mean percentage of cell lysis in samples with anti-HLA class I antibodies and serum compared with cells that were not incubated with anti-HLA Class I antibodies was 37% for the pretransplant samples and 50% for the posttransplant samples (*p* = 0.07; Figure 3). Paired analyses showed that the CD59 SABC was slightly (on average 7%) higher on the cells surviving complement-mediated cell lysis compared with living cells that were not subjected to complement-mediated cell lysis (*p* = 0.04) and also increased compared with living cells that were subjected to anti-HLA class I antibodies and HI serum (*p* = 0.02). Both pre- and posttransplantation, there was no linear correlation between the degree of cell lysis and CD59 expression on cells surviving complement-mediated cells lysis (*R* = −0.48, *p* = 0.16 and *R* = 0.12, *p* = 0.60).

### Decreased CD59 Expression following LTx Is Observed on All Lymphocyte Subsets

**Figure 4:**
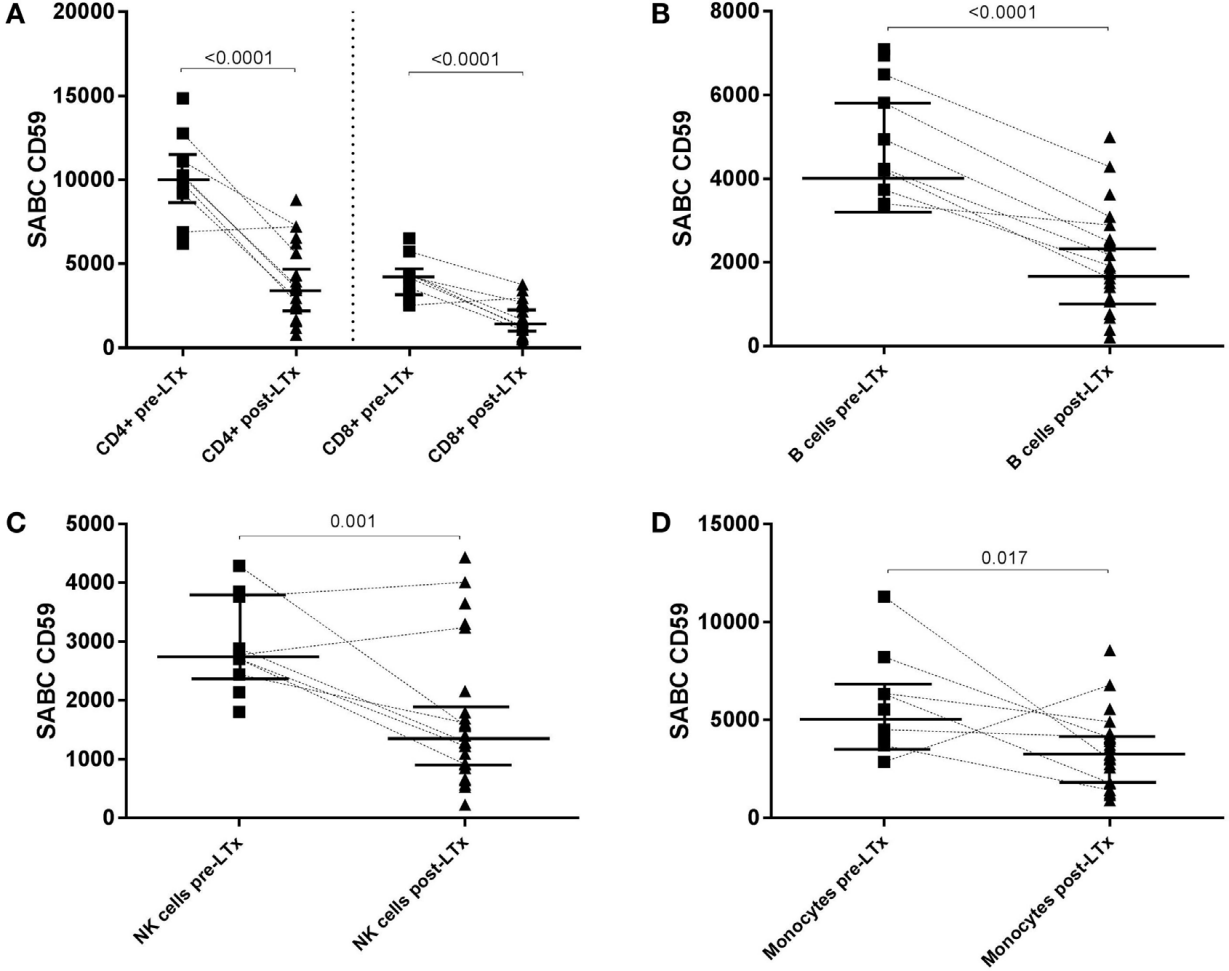
CD59 expression diminishes on all subsets following transplantation. Specific antibody-binding capacity (SABC) of CD59 on CD4+ and CD8+ T cells **(A)**, B cells **(B)**, NK cells **(C)**, and monocytes **(D)** pre- and posttransplantation was measured by flow cytometry analysis. Matched pre- and posttransplant samples are connected by a dashed line. Unpaired analyses show that the expression of CD59 is significantly lower on all subsets following transplantation (*p* < 0.05; shown in this figure). Data represent median and interquartile range; symbols indicate individual values. Data were analyzed with Mann–Whitney test.

Comparison of absolute CD59 expression on distinct lymphocyte subsets and monocytes between pre- and posttransplant samples indicated that CD59 expression decreases significantly on all subsets following LTx (Figures 4A–D). Paired sample analyses (*n* = 7) showed a similar and statistical significant pattern for CD4+ and CD8+ T cells (*p* = 0.03) and B cells (*p* = 0.02). For NK cells and monocytes, a similar decline was observed for the vast majority of patients; however, this was not statistically significant (*p* = 0.078 and *p* = 0.11, respectively). A single patient showed increased CD59 expression levels on all subsets, except for B cells, following transplantation. For this particular patient, an infectious episode at time of posttransplant sampling could not be ruled out because of limited clinical data.

**Figure 5:**
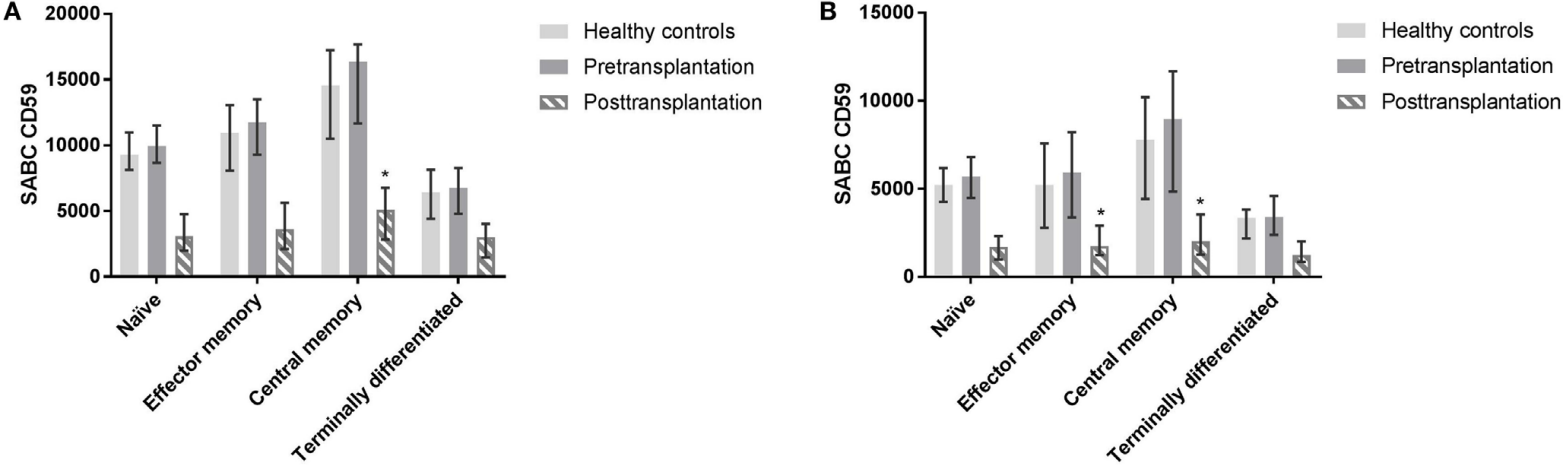
Profiling of CD59 expression on CD4+ and CD8+ T cell subsets. CD4+ and CD8+ T cells subsets were distinguished as naïve (CD45RO−CD27+), central memory (CD45RO+CD27+), effector memory (CD45RO+CD27−), and terminally differentiated T cells (CD45RO−CD27−). Posttransplantation, CD4+ central memory cells show higher expression compared with terminally differentiated (*p* = 0.007) and naïve subsets (*p* = 0.027) **(A)**. In CD8+ T cells, central and effector memory cells show higher expression compared with terminally differentiated CD8+ T cells (*p* = 0.006 and *p* = 0.027) posttransplantation **(B)**. Pretransplant samples and from healthy controls show similar expression patterns among subsets (not tested). Data represent median and interquartile range. Data were analyzed with Mann–Whitney test.

The proportion of different subsets as part of total leukocytes did not differ significantly pre- and posttransplantation. Six out of the 10 patients that were included in pretransplant measurements were on prednisone maintenance therapy before transplantation; CD59 expression on different leukocyte subsets did not differ between patients on steroid maintenance therapy or not (data not shown). Post LTx, CD4+ T cells and monocytes showed a significantly higher CD59 expression compared with CD8+ T cells, B cells, and NK cells (*p* ≤ 0.002). In CD4+ T cell subsets, central memory cells showed a significantly higher CD59 expression compared with naïve (*p* = 0.027) and terminally differentiated cells (*p* = 0.007) (Figure 5A). For CD8+ T cells, terminally differentiated cells showed a significantly lower expression of CD59 compared with central memory (*p* = 0.006) and effector memory cells (*p* = 0.027) (Figure 5B). As indicated in Figure 5 as a reference, healthy controls and pretransplant samples showed comparable expression patterns among T cell subsets.

## Discussion

CD59 plays a pivotal role in protecting cells from complement-mediated lysis and as such is considered to play an important role in the process of accommodation following organ transplantation (14, 18, 19). Yet, little is known on the expression of CD59 posttransplantation. In this study, we show that the expression of CD59 on leukocytes in lung transplant patients decreased after transplantation on all subsets. This appears not to be a general effect since the expression of other surface markers remained stable or tended to be higher following transplantation. Moreover, the expression on leukocytes of lung transplant recipients posttransplantation is also significantly lower compared with healthy controls.

We hypothesized that the observation that CD59 declines posttransplantation on all subsets may be partly attributed to immunosuppressive therapy. The expression of CD59 is mainly regulated by NF-κB and cAMP response element-binding protein (CREB) (20). It has been reported that tacrolimus, MMF, and prednisone inhibit NF-κB and CREB activity and thereby they may result in decreased CD59 transcription (21–24). However, following 24-h incubation of whole blood samples from healthy controls with these immunosuppressive drugs, we did not observe a marked decline in CD59 expression. Nor did we observe any differences in CD59 expression levels pretransplantation between patients on prednisone maintenance therapy or not. Next, we considered that the degree of inflammation at 2 months posttransplantation and further onward would be lower compared with pretransplantation when patients are hypoxic and suffer from recurrent infections and that this may have attributed to the declined CD59 expression. Several studies have shown that CD59 expression is increased at time of inflammation (25–27). However, stimulation of PBMCs with CD3/CD28 dynabeads did only result in a moderate increase in CD59 expression. Therefore, additional causes for the lowered CD59 expression following LTx including the production of autoantibodies directed against cell self-antigens (28, 29), consumption of CD59, complement-mediated lysis (30, 31), and shedding should be considered (32, 33).

Because of the already extensive antibody panel and limited cell numbers, we could not quantify the expression of the other membrane-bound complement-regulating proteins CD46 and CD55 in this study. Given our findings, it would be of interest to investigate in future studies whether the expression levels of these latter proteins also decline following transplantation. The use of mass cytometry (CyTOF) instead of flow cytometry may overcome the issue of limited color detection possibilities. Another limitation is that we could not determine whether CD59 expression levels on endothelial cells posttransplantation also decline because transbronchial biopsies are not routinely being performed in our center. Furthermore, because of the strong expression of CD59 on endothelial cells, we can only estimate the antigen density on these large cells. Perhaps this could also be resolved by using CyTOF.

The highest expression of CD59, both pre- and posttransplantation, was observed on CD4+ T cells and monocytes. To the best of our knowledge, this is the first study to report quantitative expression levels of CD59 on different leukocyte subsets. Rao et al. previously reported that in healthy subjects the MFI was higher on myeloid cells including monocytes compared with lymphocytes (13). Others showed that, like in our study, the relative expression is lower on NK cells compared with T cells (12) and within T lymphocytes CD45RO+ T cells show a higher expression compared with CD45RO− T cells (12, 34). CD59 serves to downregulate CD4+ and CD8+ T cell proliferation and activation upon antigen recognition through binding of CD59 with its ligand on antigen-presenting cells (8, 34, 35). For monocytes, it has been postulated that they may particularly benefit from high CD59 expression because of their phagocytic function at inflammatory sites featuring vigorous complement activation (36).

Following the same lysis protocol, there was a trend toward a higher mean percentage of lysis for the posttransplant samples compared with pretransplantation. Also the expression of CD59 on cells surviving lysis was slightly higher compared with the cells that were not subjected to complement-mediated cell lysis. However, we could not observe a linear correlation between percentage of overall cell lysis and absolute CD59 expression on leukocytes. The lowered CD59 expression on leukocytes posttransplantation may also have complement-independent effects involving altered cell signaling like enhanced T-cell activation upon alloantigen presentation as mentioned earlier (8, 34, 35). In NK cells, CD59 enhances killing *via* interaction with natural cytotoxicity receptors (10, 37). For monocytes and B cells, no direct complement-independent roles for CD59 have been described thus far.

We hypothesize that similar mechanisms as in leukocytes may potentially also lead to lowered CD59 expression on endothelial cells within the allograft because of complement activation or shedding. Given the high expression of CD59 on lung donor endothelial cells compared with PBMCs, we hypothesize that this may not necessarily alter sensitivity to complement-mediated cell lysis but could rather favor a procoagulant and proinflammatory phenotype (4, 7). Supporting this hypothesis, we have previously reported that endothelial cells with a genotype that is associated with a lower CD59 expression secrete higher levels of fibroblast growth factor β and interleukin-6 upon exposure to sublytic complement (17).

In summary, we show that CD59 expression on leukocytes is significantly lower in lung transplant patients compared with healthy controls and patients with end-stage lung disease. This lowered expression following LTx is observed on all distinct lymphocyte subsets and monocytes. This lowered CD59 expression could be the result of complement activation or shedding of CD59. This study opens new perspective for further research to elucidate the mechanisms behind this lowered CD59 expression and to investigate whether these mechanisms also affect CD59 expression on the donor endothelium.

## Ethics Statement

All patients gave written informed consent in accordance with the Declaration of Helsinki. The protocol was approved by the institutional review board (Medisch Ethische Toetsingscommissie) of the UMC Utrecht (protocol METC 06-144).

## Author Contributions

DD, TK-H, and LM performed the research; KB, DD, TK-H, LM, HO, MV, and AZ participated in data analysis; EG contributed patient material; KB, EG, LM, and HO participated in research design; KB, LM, HO, MV, and AZ wrote the paper. All the authors provided final approval of the version to be published.

## Conflict of Interest Statement

AZ has received a travel grant and/or speakers fee from Astellas Pharma and Alexion and is on the advisory board of Novartis. EG and LM have received a travel grant from Astellas Pharma. All other authors have no conflict of interest to disclose.

## Supplementary Material

Figure S1 Gating strategy. Leukocytes subsets were identified based on FSC/SSC and CD45 expression and are further characterized based on CD3. T cells are selected from the CD45+CD3 +gate and differentiated as CD4+ and CD8+ T cells. CD4+ and CD8+ T cell subsets were distinguished as naïve (CD45RO−CD27+), central memory (CD45RO+CD27+), effector memory (CD45RO+CD27−), and terminally differentiated T cells (CD45RO−CD27−) **(A)**. B cells are defined as CD45+CD3−CD19+ cells and NK cells as CD45+CD3−CD16+CD56+ **(B)**. Finally classical monocytes were selected based on CD45+CD3−C14+CD16− expression and on their FSC/SSC **(C)**.

Figure S2 Proportion of different leukocyte subsets over time posttransplantation. Percentage of different leukocyte subsets stratified according to different sampling times posttransplantation. Data represent mean and standard error of the mean.

Figure S3 Estimated CD59 expression on endothelial cells is notably higher compared with leukocytes. Depiction of specific antibody-binding capacity (SABC) of CD59 on leukocytes calculated by using the QIFIKIT on the left *y*-axis and estimated SABC of CD59 on lung donor endothelial cells based on anti-CD59 PE median fluorescence intensity calculated by using Quantibrite™ beads on the right *y*-axis. Data represent median and interquartile range; symbols indicate individual values.

## Abbreviations

ABC, antibody-binding capacity; BOS, bronchiolitis obliterans syndrome; CREB, cAMP response element-binding protein; LTx, lung transplantation; MAC, membrane attack complex; mCRP, membrane complement regulatory protein; MFI, mean fluorescence intensity; MMF, mycophenolate mofetil; NF-κB, nuclear factor-kappaB; PBMC, peripheral blood mononuclear cell; PBS, phosphate-buffered saline; SABC, specific antibody-binding capacity; SNP, single-nucleotide polymorphism.
