# Supplementary figures and images for: Reduced Expression of Membrane Complement Regulatory Protein CD59 on Leukocytes following Lung Transplantation
Source: Front Immunol. 2018 Jan 22;8:2008. doi: 10.3389/fimmu.2017.02008 (PMC5786830; doi:10.3389/fimmu.2017.02008)

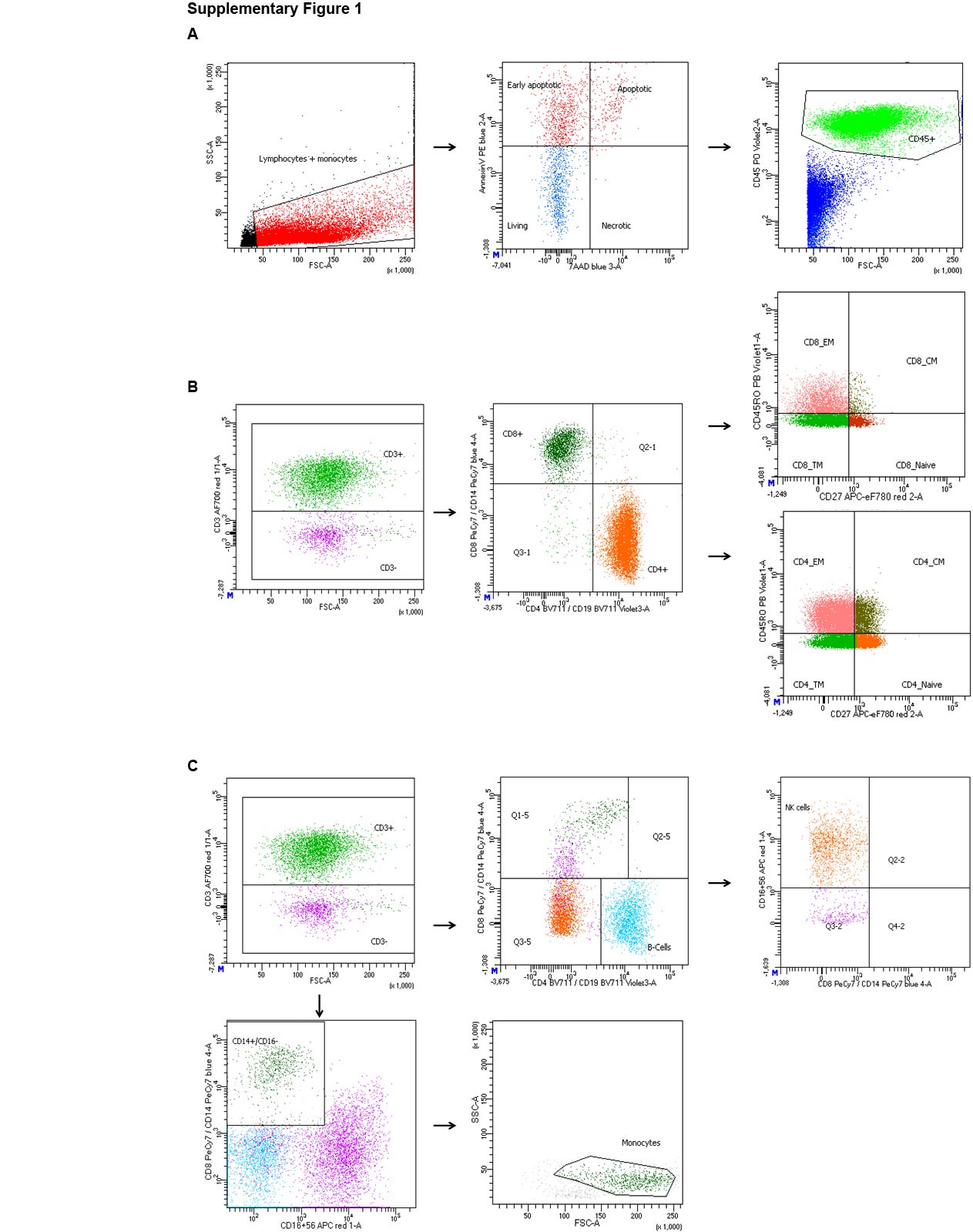

Supplement: Figure S1 — Gating strategy. Leukocytes subsets were identified based on FSC/SSC and CD45 expression and are further characterized based on CD3. T cells are selected from the CD45+CD3 +gate and differentiated as CD4+ and CD8+ T cells. CD4+ and CD8+ T cell subsets were distinguished as naïve (CD45RO−CD27+), central memory (CD45RO+CD27+), effector memory (CD45RO+CD27−), and terminally differentiated T cells (CD45RO−CD27−) (A). B cells are defined as CD45+CD3−CD19+ cells and NK cells as CD45+CD3−CD16+CD56+ (B). Finally classical monocytes were selected based on CD45+CD3−C14+CD16− expression and on their FSC/SSC (C). [file image_1.jpeg]

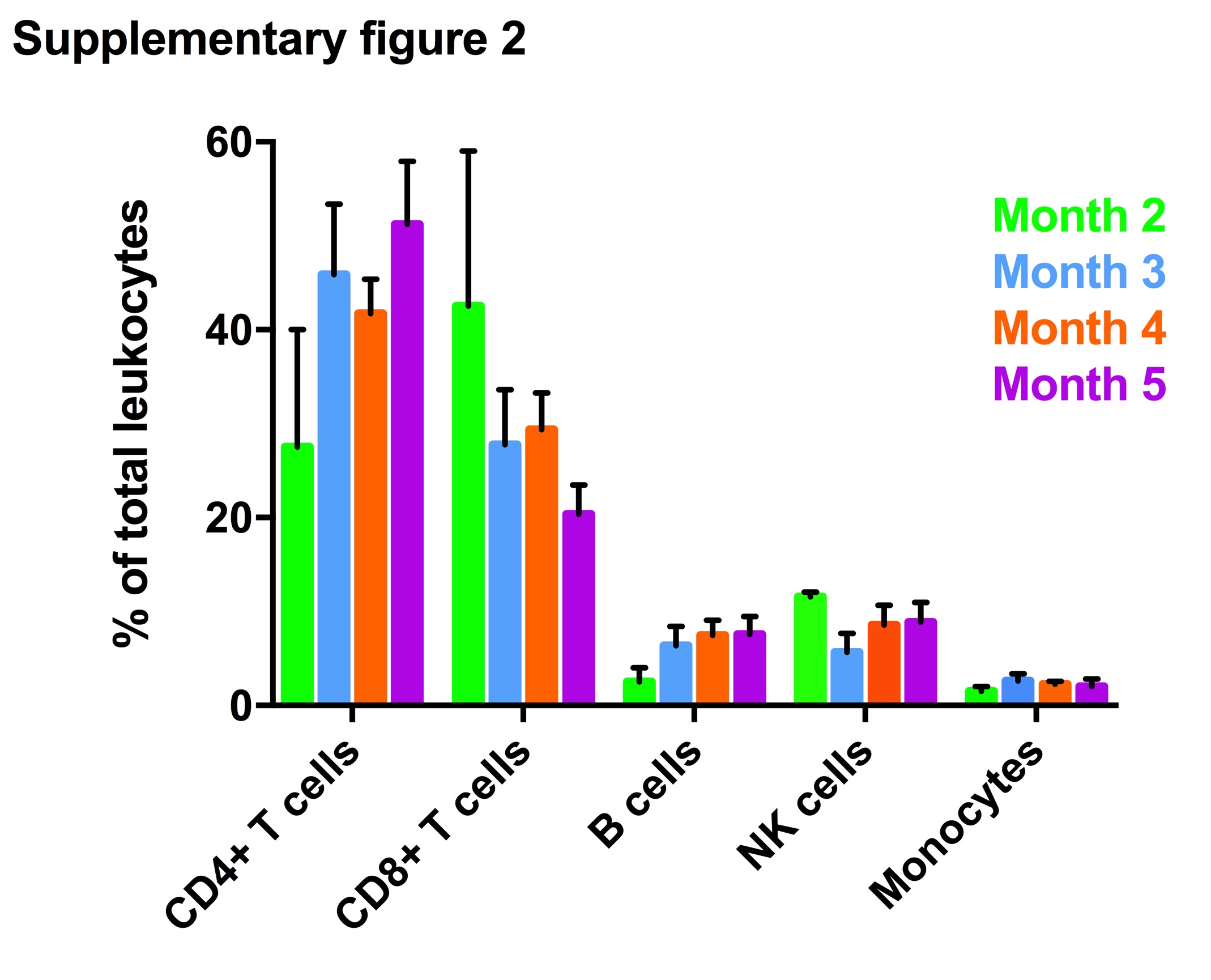

Supplement: Figure S2 — Proportion of different leukocyte subsets over time posttransplantation. Percentage of different leukocyte subsets stratified according to different sampling times posttransplantation. Data represent mean and standard error of the mean. [file image_2.jpeg]

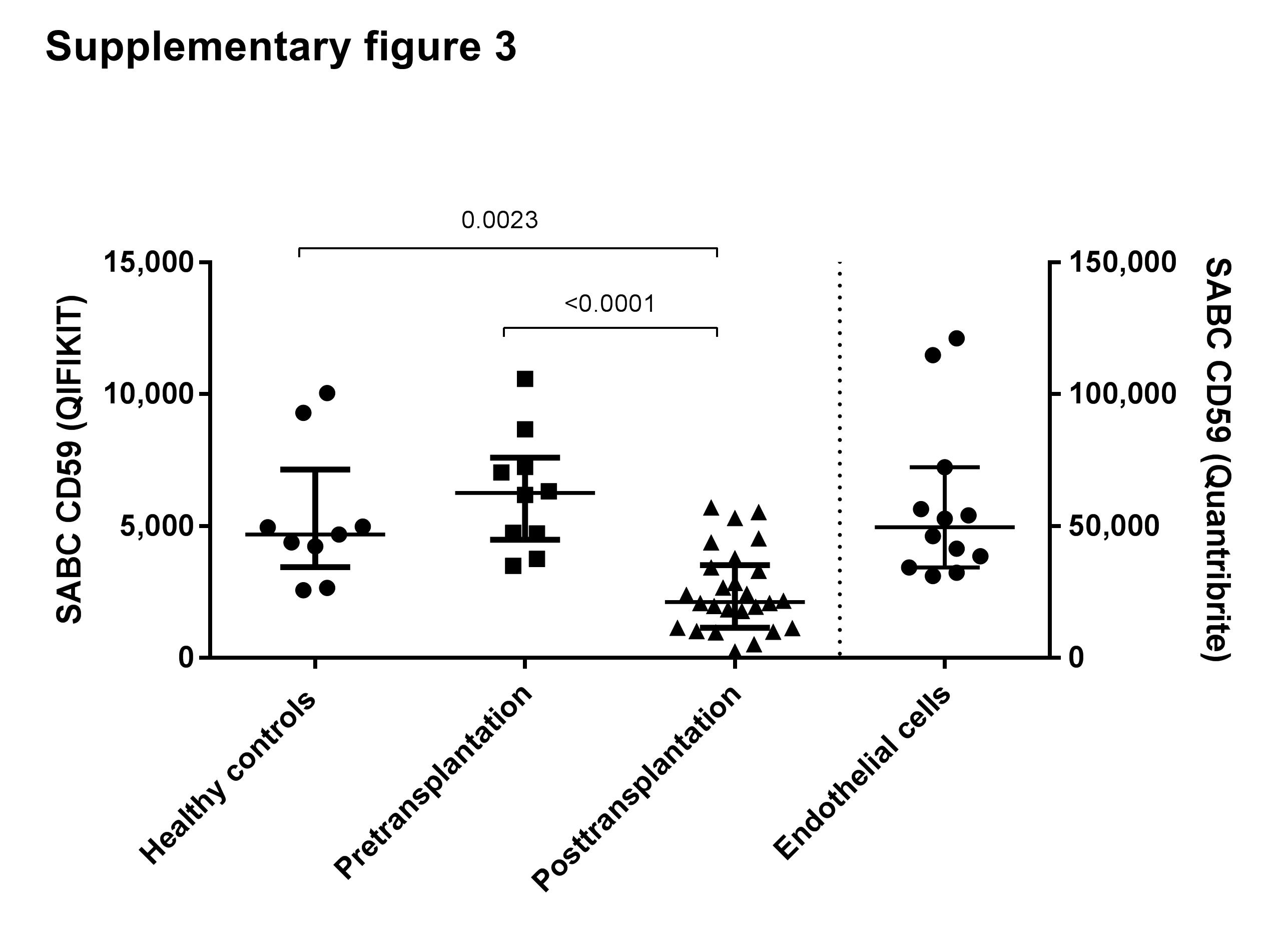

Supplement: Figure S3 — Estimated CD59 expression on endothelial cells is notably higher compared with leukocytes. Depiction of specific antibody-binding capacity (SABC) of CD59 on leukocytes calculated by using the QIFIKIT on the left y-axis and estimated SABC of CD59 on lung donor endothelial cells based on anti-CD59 PE median fluorescence intensity calculated by using Quantibrite™ beads on the right y-axis. Data represent median and interquartile range; symbols indicate individual values. [file image_3.jpeg]
