# Supplementary material for: Reduced Expression of Membrane Complement Regulatory Protein CD59 on Leukocytes following Lung Transplantation
Source: Front Immunol. 2018 Jan 22;8:2008. doi: 10.3389/fimmu.2017.02008 (PMC5786830; doi:10.3389/fimmu.2017.02008)
Supplement: Supplementary file 4 [file data_sheet_1.docx]

**Supplementary methods**

In order to relate CD59 expression on leukocytes to endothelial cells, we determined the specific antibody binding capacity (SABC) on lung donor endothelial cells obtained at time of transplantation (method for isolation has previously been described^[[1]](#footnote-1)^). Because of the high CD59 expression on endothelial cells, cells were incubated with a mixture of 4μl 50ng/ml anti-CD59 PE (BD Bioscience, San Diego, CA) and 9μl 500ng/ml unlabeled anti-CD59 (Biolegend, San Diego, CA) against the same epitope. The SABC based on the anti-CD59 PE median fluorescence intensity was determined by using Quantibrite beads (BD) with bead populations with distinct amount of PE molecules, in order to avoid a secondary staining step with goat anti-mouse IgG FITC as used in the QIFIKIT. Since we performed competitive staining with anti-CD59 PE and unlabeled anti-CD59, the actual CD59 SABC on endothelial cells will be even higher.

1. Budding K, van de Graaf EA, Kardol-Hoefnagel T, Broen JCA, Kwakkel van Erp JM, Oudijk EJD, van Kessel DA, Hack CE, Otten HG. A promoter polymorphism in the CD59 complement regulatory protein gene in donor lungs correlates with a higher risk for chronic rejection after lung transplantation. *Am J Transplant* (2016) 16:987–98. doi:10.1111/ajt.13497 [↑](#footnote-ref-1)
